# CT and MRI Characterization of a Retroperitoneal Lymphatic Malformation in an Adult

**DOI:** 10.7759/cureus.92002

**Published:** 2025-09-10

**Authors:** Jochen Gerstner Saucedo, Yudel Tamayo, Mohammad Saleh, Beatrice Luiza Madrazo

**Affiliations:** 1 Diagnostic Radiology, University of Colorado Anschutz Medical Campus, Aurora, USA; 2 Diagnostic Radiology, University of Miami Miller School of Medicine/Jackson Memorial Hospital, Miami, USA; 3 Radiology, Jackson Memorial Hospital, Miami, USA; 4 Radiology, University of Miami Miller School of Medicine, Miami, USA

**Keywords:** adult abdominal mass, cystic lesion, lymphatic malformation, multimodal imaging, retroperitoneal cyst, vascular anomaly

## Abstract

Lymphatic malformations (LMs) of the abdomen are uncommon congenital vascular anomalies that typically present during childhood. The nonspecific symptoms and radiological overlap with other cystic lesions may present significant diagnostic challenges, as their occurrence in adults is uncommon. We present the case of a 19-year-old male patient with human immunodeficiency virus (HIV) on antiretroviral therapy (ART), who presented with lower abdominal pain and fullness. Computed tomography (CT) imaging revealed a large, retroperitoneal, multiloculated cystic mass characterized by low attenuation and internal septations, resulting in bowel displacement. Magnetic resonance imaging (MRI) further characterized the lesion as complex and cystic, with additional features consistent with a benign vascular anomaly. The diagnosis of lymphatic malformation was confirmed through histopathological examination following a CT-guided biopsy. The patient experienced an uncomplicated recovery following the surgical resection of the lesion. This case highlights the significance of multimodal imaging, specifically how CT and MRI complement each other, in the precise diagnosis of a lymphatic malformation and the enhancement of the differential diagnosis.

## Introduction

Intra-abdominal lymphatic malformations (LMs), previously referred to as cystic lymphangiomas, are uncommon benign vascular anomalies arising from the lymphatic system [1,2]. LMs originate from congenital errors in the embryologic development of the lymphatic system, resulting in sequestration and abnormal proliferation of lymphatic endothelial cells that fail to establish normal connections with the central lymphatic or venous circulation. This condition leads to the development of cystic or cavernous lymphatic channels within the abdominal cavity, most frequently observed in the mesentery, omentum, or retroperitoneum. Recent molecular studies have found that somatic mutations, especially in genes such as PIK3CA, contribute to the condition's development by promoting abnormal growth of lymphatic endothelial cells and lymphangiogenesis [3,4].

Their presence in adults is rare, which emphasizes their unique nature and frequently makes the diagnosis challenging due to nonspecific symptoms and imaging features that overlap with other cystic lesions [5,6]. The overall incidence of lymphatic malformations (across all anatomical sites) is estimated to be 1.2-2.8 per 100,000 children; however, specific incidence rates for intra-abdominal forms are not well established due to their rarity and frequent incidental discovery [7]. These malformations are most frequently identified incidentally or during diagnostic evaluations for symptoms such as abdominal pain, distension, or palpable masses. Conversely, acute presentations resulting from complications such as infection, hemorrhage, or bowel obstruction are less common [1,2].

Imaging plays an important role in identifying characteristic features of LMs, such as a well-circumscribed, multiloculated cystic mass with thin enhancing septations and homogeneous fluid content. Mild enhancement of the cyst wall or septa may be seen, and the lesions are typically non-invasive but can cause mass effect on adjacent structures [1]. Magnetic resonance imaging (MRI) is especially valuable for non-invasive characterization and distinguishing these lesions from other cystic abdominal masses; however, a definitive diagnosis continues to require histopathological confirmation [5,6,8].

On histopathology, these malformations are composed of dilated lymphatic channels lined by a single layer of flattened endothelial cells within a fibrous stroma. Immunohistochemical staining is crucial for diagnosis, with D2-40 (podoplanin) and CD31 being the most frequently employed markers to verify lymphatic endothelial origin. The diagnosis is often substantiated only following surgical excision and histopathological assessment, owing to the nonspecific nature of clinical and radiological characteristics [9].

Given the rarity of this condition in adults, initial management should be symptomatic and tailored to the lesion localization and associated complications if present. However, a definitive diagnosis always requires histopathological analysis to confirm the diagnosis [10,11].

## Case presentation

A 19-year-old male patient with a past medical history of human immunodeficiency virus (HIV) on antiretroviral therapy (ART) presented to the emergency department with three days of lower abdominal pain and a sensation of fullness. The patient denied associated symptoms such as fever, nausea, or weight loss. During the physical examination, vital signs were within normal limits. Abdominal examination showed mild tenderness in the left lower quadrant without guarding, rebound, or palpable mass, and there were no signs of peritonitis or systemic toxicity. The rest of the physical examination was unremarkable.

**Figure 1:**
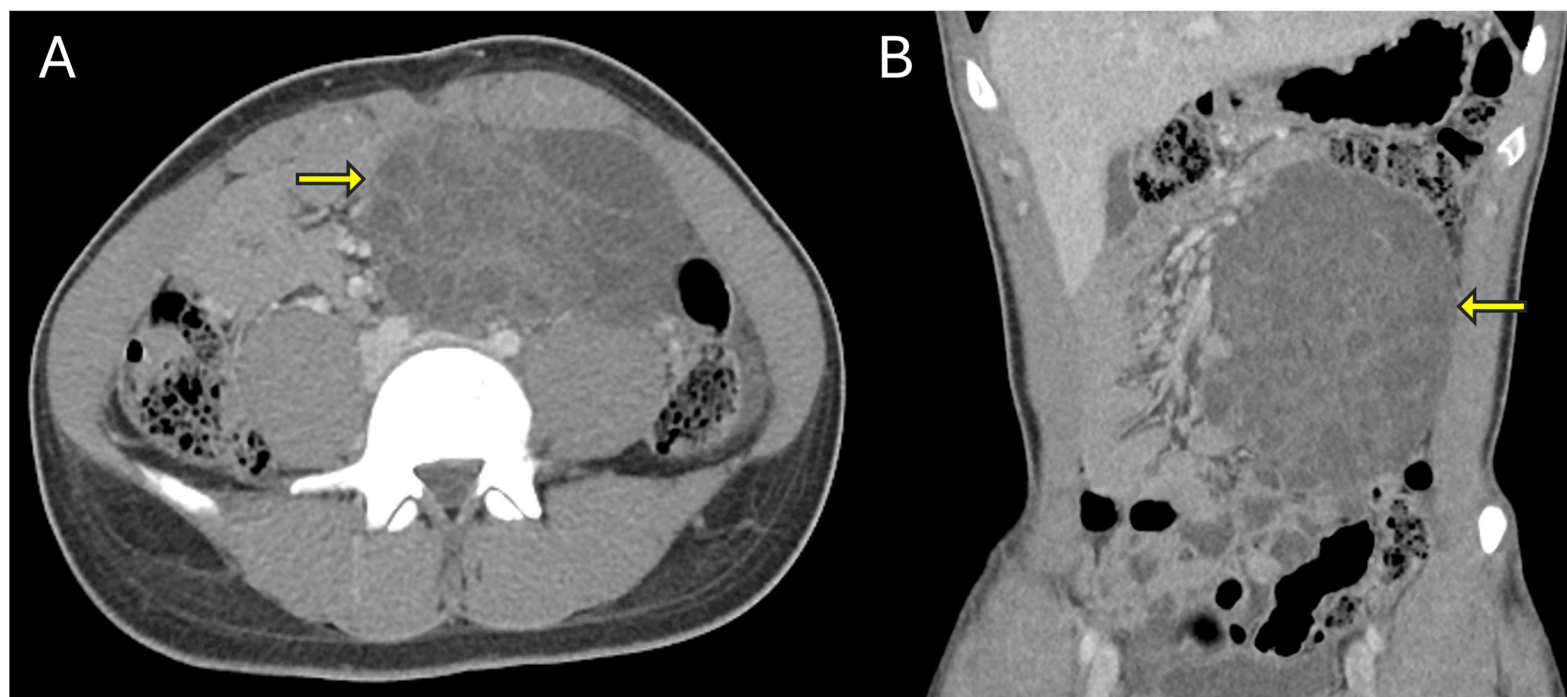
Axial (A) and coronal (B) contrast-enhanced CT images of the abdomen demonstrating a large, well-defined, multiloculated cystic mass (yellow arrows) occupying the left retroperitoneal space. The lesion displays low attenuation with internal septations and mass effect on adjacent bowel loops without evidence of obstruction. CT: computed tomography

The initial workup included a computed tomography (CT) scan of the abdomen and pelvis with intravenous (IV) contrast, which revealed a large, heterogeneous, multiloculated cystic mass in the left abdomen, measuring approximately 12.7 × 7.3 × 12.9 cm. The lesion showed low attenuation with septations and was associated with the displacement of adjacent bowel loops without any sign of obstruction. The differential diagnosis included inflammatory myofibroblastic tumor, lymphatic malformation, mesenteric fibromatosis, lymphoma, or other mesenchymal tumors (Figure 1).

**Figure 2:**
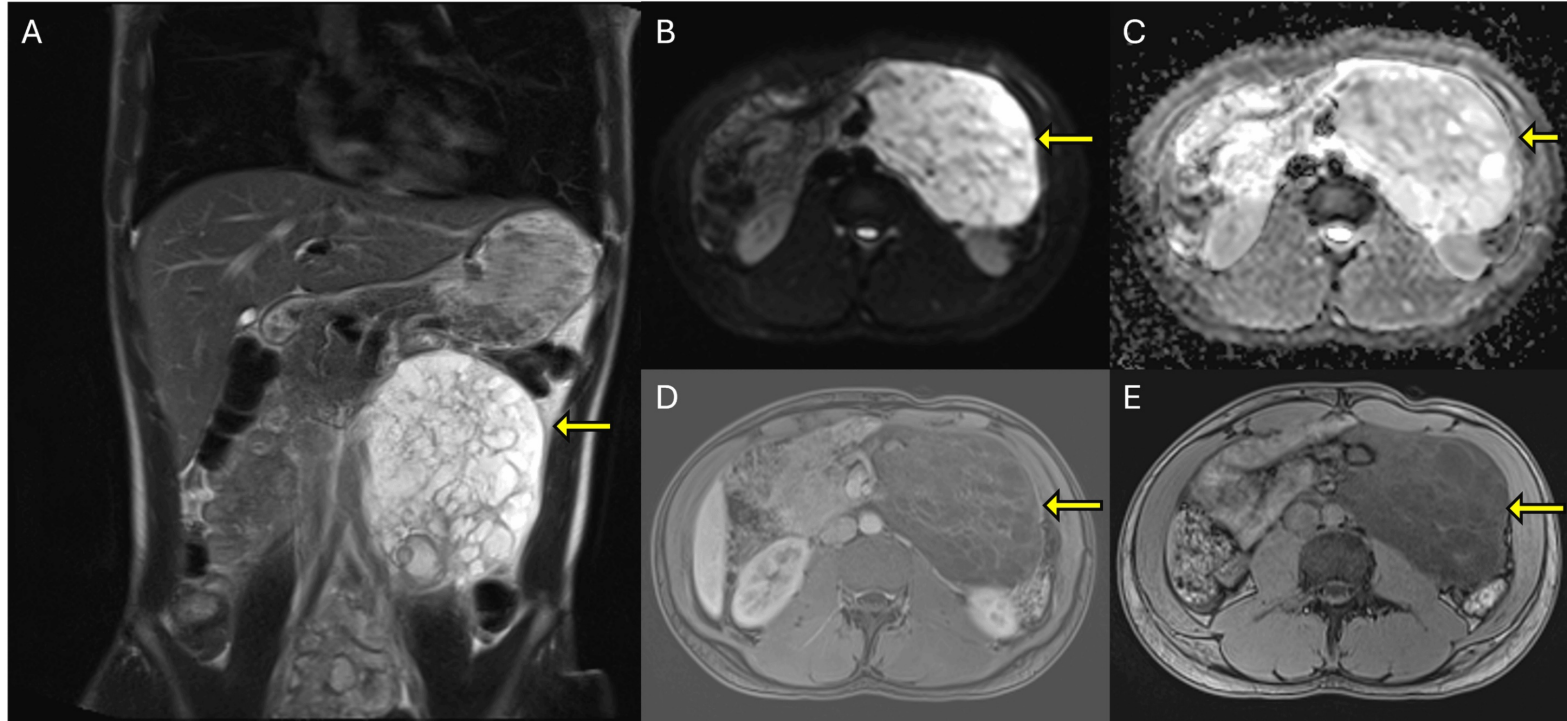
MRI characterization of the abdominal mass. (A) Coronal T2-weighted image demonstrates a large, multiloculated, hyperintense mass with internal septations in the left abdomen. (B) DWI shows high signal intensity within the lesion. (C) Corresponding ADC map demonstrates no true diffusion restriction, consistent with T2 shine-through. (D) Axial post-contrast fat-suppressed T1-weighted image shows mild enhancement of internal septations without solid enhancing components. (E) Axial T1-weighted image shows the lesion as hypointense relative to the surrounding soft tissues. MRI: magnetic resonance imaging, DWI: diffusion-weighted imaging, ADC: apparent diffusion coefficient

**Figure 3:**
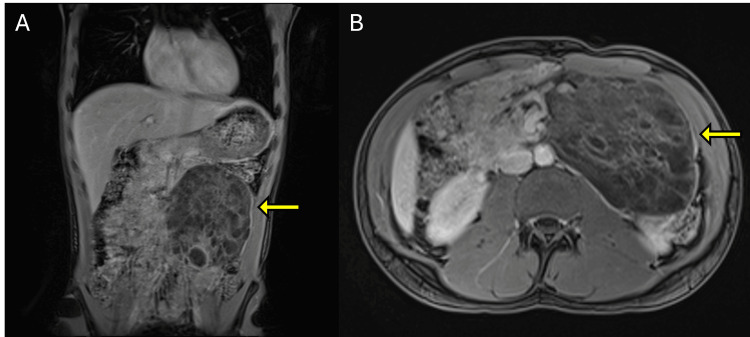
(A) Coronal and (B) axial T1-weighted post-contrast MRI images in the portal venous phase showing a large, multiloculated cystic mass in the left retroperitoneum (yellow arrows). The lesion demonstrates thin, mildly enhancing internal septations without solid enhancing components. These findings are consistent with a macrocystic lymphatic malformation. MRI: magnetic resonance imaging

MRI of the abdomen confirmed a large, complex, septated, T2-hyperintense mass with no solid components and variable internal signal, suggestive of proteinaceous or chylous content (Figure 2). Post-contrast T1-weighted sequences acquired in the portal venous phase demonstrated thin, mildly enhancing septations without nodular or solid enhancement, further supporting the diagnosis of a macrocystic lymphatic malformation (Figure 3).

The patient underwent image-guided core needle biopsy under CT guidance. Grossly, the mass contained a viscous, milky fluid. Cytology showed lymphocytes and macrophages, and surgical pathology revealed irregular vascular spaces lined by endothelium positive for D2-40 and CD34, consistent with a diagnosis of lymphatic malformation. Immunohistochemical stains excluded lymphoma, with no morphologic features of malignancy observed. Although histopathological confirmation was obtained, corresponding images were unavailable for inclusion in this report.

Due to the size of the mass, the patient's ongoing symptoms, and diagnostic uncertainty, surgical resection of the mass was performed. Intraoperatively, a 12 x 12 x 8 cm retroperitoneal tumor was identified and excised. Final pathology confirmed lymphatic malformation with associated secondary fibroinflammatory changes. Postoperatively, the patient recovered without complications and was discharged with follow-up to be scheduled as necessary by his primary medical team.

## Discussion

Lymphatic malformations (LMs) are a range of congenital vascular anomalies caused by abnormal lymphatic development. In the abdomen, these lesions most frequently affect the mesentery, omentum, or retroperitoneum. Their incidence in adults is not well established and is primarily inferred from case reports in the literature, with many adult cases found incidentally or after symptoms such as abdominal discomfort, distension, or appearance of a mass effect [1,2]. Due to its rarity, particularly in the adult population, most available data come from isolated case reports and small case series, and no reliable prevalence by age, ethnicity, or geographic region has been established.

Cross-sectional imaging is essential for characterizing these lesions. On CT, intra-abdominal LMs usually appear as well-defined, hypodense, multiloculated cystic masses with thin, enhancing borders and septations [5,6]. MRI provides further detail of the lesion's content, showing T2-hyperintensity with variable T1 signals depending on the presence of hemorrhagic or chylous components [5,12]. Atypical imaging features, such as septal calcifications, unilocular appearance, or heterogeneous contents, can obscure diagnosis and expand the differential, which includes mesenteric cysts, cystic mesotheliomas, pseudocysts, enteric duplication cysts, and cystic neoplasms such as mucinous cystic tumors [5,13]. Table 1 summarizes the imaging features relevant to the differential diagnosis of lymphatic malformations, comparing them to other cystic abdominal lesions.

**Table 1:** Imaging features of lymphatic malformations and other cystic abdominal lesions

Lesion	Typical location	Locularity	Wall/septal characteristics	Enhancement	Other key features	References
Lymphatic malformation	Mesentery, retroperitoneum	Multilocular	Thin walls and septa	Minimal or no enhancement	Homogeneous fluid; absence of solid components or calcifications	[5,6,14]
Cystic mesothelioma	Pelvic peritoneum, omentum	Multilocular	Thin septations; may be irregular	Mild septal enhancement	Associated with prior surgery or inflammation; peritoneal distribution	[6,14,15]
Pancreatic pseudocyst	Peripancreatic region	Unilocular	Thicker wall; may contain debris	Variable; typically wall enhancement	History of pancreatitis; lacks septations; adjacent to pancreas	[6,14]
Mesenteric cyst	Mesentery	Unilocular or few septa	Thin wall, clear fluid	Minimal	Less multilocular than lymphatic malformations; often incidental	[6,16]
Enteric duplication cyst	Adjacent to the bowel wall	Unilocular	Double-layered wall (muscularis and mucosa)	Inner lining may enhance	May contain proteinaceous or hemorrhagic content; bowel continuity	[6,14]
Mucinous cystic neoplasm	Ovary or pancreas	Unilocular or multilocular	Thick wall/septa; mural nodules possible	Wall/septa and nodules may enhance	Internal complexity; solid components; potential for malignancy	[6,14]

Management strategies for LMs depend on the lesion's size, clinical symptoms, anatomical location, and potential risks. Complete surgical excision remains the preferred standard for lesions that cause symptoms, are growing, or are diagnostically unclear, providing both treatment and diagnostic information [1,11]. Nonetheless, percutaneous sclerotherapy, a less invasive procedure where a sclerosing agent is injected into the cyst, has become an effective alternative, especially for macrocystic lesions or in patients for whom surgery poses high risks [2,10]. Although the recurrence rate is generally low following complete excision, it may be elevated if only a portion of the lesion is removed or in instances of multifocal disease [17].

Histopathological examination remains essential for confirming the diagnosis. Lymphatic malformations (LMs) consist of dilated lymphatic channels lined by flattened endothelium, frequently accompanied by adjacent fibrous stroma and occasional hemorrhage or inflammation [8]. Immunohistochemical analysis typically reveals positivity for D2-40 (podoplanin), a marker indicative of lymphatic endothelial cells, and may also demonstrate reactivity for CD31 (an endothelial cell marker) and vascular endothelial growth factor receptor 3 (VEGFR3), all of which substantiate the lymphatic origin of the lesion [18].

## Conclusions

Intra-abdominal lymphatic malformations are uncommon in adults and can mimic various cystic lesions on imaging. This case emphasizes the importance of multimodal imaging in diagnosis, where CT provides anatomical details and initial assessment, and MRI offers enhanced tissue contrast, septal visualization, and confirmation of benign features through contrast enhancement and diffusion sequences. Recognizing these imaging characteristics can strongly suggest the diagnosis before surgery, assist in guiding biopsies or surgical planning, and help prevent misdiagnosis. Radiologists and clinicians should keep lymphatic malformations in mind when diagnosing large, multiloculated cystic abdominal masses in adult patients, while also considering other cystic lesions with overlapping imaging features as part of the differential diagnosis.
